# European Reference Network (ERN) ReCONNET methodology for the cross-cultural adaptation of instruments for research and care in the context of rare connective tissue diseases (CROSSADAPT)

**DOI:** 10.1186/s13023-025-03674-8

**Published:** 2025-05-15

**Authors:** Laurent Arnaud, Oliver Sander, Simona Rednic, Philippe Mertz, Raquel Faria, Francesca Crisafulli, Sofia Silva-Ribeiro, Lou Kawka, Cedric Sztejkowski, Christina Düsing, Thomas Rose, Antonio Lamas, Carlos Vasconcelos, Giulia Fontana, Paolo Semeraro, Teodora Neagu, Mihaela Resteu, Laura Damian, Cristina Pamfil, Camelia Bucsa, Lisa J. Matthews, Rosaria Talarico, Marta Mosca, Giuseppe Turchetti, Thomas Thibault, Hervé Devilliers

**Affiliations:** 1 Department of Rheumatology, National Reference Center for Rare Autoimmune Diseases (RESO), Hôpitaux Universitaires de Strasbourg, Strasbourg, France; 2 Department for Rheumatology and Hiller Research Centre for Rheumatology, University Hospital, Düsseldorf, Germany; 3 Department of Rheumatology, Emergency County Teaching Hospital, University of Medicine and Pharmacy Iuliu Hatieganu, Cluj-Napoca, Romania; 4 Department of Internal Medicine, National Reference Center for Autoinflammatory Diseases and Inflammatory Amyloidosis (CEREMAIA), Tenon Hospital, APHP, Paris, France; 5 Unidade de Imunologia Clínica, Hospital de Santo António, Unidade Local de Saúde Santo António, Porto, Portugal; 6 Rheumatology and Clinical Immunology Unit, ASST Spedali Civili of Brescia, Department of Clinical and Experimental Sciences, University of Brescia, Brescia, Italy; 7 Porto, Portugal; 8 Klinik Für Rheumatologie Und Klinische Immunologie, Charité Universitätsmedizin Berlin, Charité Platz 1, 10117 Berlin, Germany; 9 ASPOR Association of Romanian Relapsing Polychondritis Patients &, Iuliu Hațieganu University of Medicine and Pharmacy, Cluj-Napoca, Romania; 10 Relapsing Polychondritis Awareness & Support, (Relapsingpolychondritis.Org), Worcester, UK; 11 Rheumatology Unit, Azienda Ospedaliero Universitaria Pisana, University of Pisa, Pisa, Italy; 12 Institute of Management, Scuola Superiore Sant’Anna, 56127 Pisa, Italy; 13 Internal Medicine and Systemic Diseases Unit, University Hospital Centre Dijon, Dijon, France

**Keywords:** Cross-cultural adaptation, Rheumatology, Rare diseases, Orphan diseases, Reference networks

## Abstract

The traditional process of intercultural adaptation, while suitable for one or a few target languages, is not optimal for developing instruments for rare connective tissue diseases (CTDs) in multiple languages simultaneously. The European Reference Network ReCONNET presents the protocol for a novel methodology for cross-cultural adaptation of instruments for research and care in the context of rare CTDs (ReCONNET-CROSSADAPT). It is initiated by the identification of ‘key-terms’ that are crucial for maintaining the original meaning of the source document. Each language group, led by a senior member and two collaborators, independently assesses the existence and equivalence of these key terms in target languages. Reconciliation meetings are held to establish agreed-upon terms for consistent usage across translations when difficulties arise with key-terms. Subsequently, each language group translates the source document, followed by a reconciliation meeting involving one CTD patient in each group. The purpose of this meeting is to address potential discrepancies among translations, ensuring a comprehensive assessment from a linguistic, cultural and patient perspective. Collective feedback and consensus-based decision-making guide the resolution process. This methodology eliminates the need for backward translation, optimizing time and cost utilization. This new ReCONNET-CROSSADAPT methodology ensures linguistic accuracy, cultural relevance, and contextual appropriateness for the cross-cultural adaptation of instruments for research and care in the context of rare CTDs.

**Supplementary Information:**

The online version contains supplementary material available at 10.1186/s13023-025-03674-8.

## Key messages

- **What is already known on this topic:** The traditional process of intercultural adaptation is complex and particularly not optimal for developing instruments for rare connective tissue diseases (CTDs) in multiple languages simultaneously.
- **What this study adds:** Here, we presents a new methodology (ReCONNET-CROSSADAPT) for cross-cultural adaptation of instruments for research and care in the context of rare CTDs, relying upon the identification of key-terms, translation by knowledgeable clinicians and reconciliation involving patients with the disease.
- **How this study might affect research, practice or policy:** The ReCONNET-CROSSADAPT methodology ensures linguistic accuracy, cultural relevance, and contextual appropriateness for the cross-cultural adaptation of instruments for research and care in the context of rare CTDs.

**Supplementary Information:**

The online version contains supplementary material available at 10.1186/s13023-025-03674-8.

## Introduction

European Reference Networks (ERNs) are virtual networks involving healthcare professionals (HCPs) across Europe. Their aim is to tackle rare and complex diseases and conditions that require highly specialized treatment and a concentration of knowledge and resources [1]. The ERN on Connective Tissue and Musculoskeletal Diseases (ReCONNET), involves 63 Healthcare Providers from 23 European Union (EU) Member states and 15 patients’ representatives. ERNs promote activities aimed at improving the knowledge on rare and complex disease such as webinars, exchanges between expert centers, development of lay versions on clinical practice guidelines, red flags for early diagnosis, as well as the development of instruments for improving the care of patients with rare connective tissue diseases (CTDs) [1]. Here we present the protocol of the ERN ReCONNET methodology for the cross-cultural adaptation of instruments for research and care, simultaneously across several languages (CROSSADAPT). Since January 2024, the ERN ReCONNET has initiated the development of the relapsing polychondritis health-related quality of life assessment instrument (RP-QoL), which is a novel instrument for assessing disease-specific health-related QoL in patients with RP in multiple EU languages [2], using this ERN ReCONNET CROSSADAPT methodology.

Unlike translation, which focuses primarily on converting words from one language to another, cross-cultural adaptation involves deeper contextual modifications to ensure that the instrument remains meaningful and effective in different cultural contexts [3]. It aims to minimize bias and ensure the accurate capture of data from different cultural perspectives, thus enhancing the applicability and usefulness of the instrument in a variety of clinical or research contexts. More than 30 potential strategies have been proposed for cross-cultural adaptation [4]. A review of the proposed recommendations does not reveal any unanimously recognized consensus [4]. Our aim was to face the challenges of simultaneous adaptation in several languages for rare CTDs diseases with limited resource constraints, while respecting, as far as possible, the constraints of the above-mentioned milestones. In addition, since the coordination of iterative processes becomes more complex in several languages, it is, therefore, essential to adopt a standardized method, ensuring the most reproducible equivalence possible from one language to another between the source document and the original material [3]. Consequently, it is imperative to adopt a clear, integrated and coordinated approach tailored to the unique linguistic, cultural and logistical challenges of multilingual adaptation, in order to ensure the validity, reliability and cultural sensitivity of instruments in diverse linguistic contexts, as expected within the frame of the ERN ReCONNET. **The present paper describes the ERN ReCONNET methodology for the cross-cultural adaptation of instruments for research and care in the context of rare CTDs**.

### Usual methodologies for cross-cultural adaptation

**Fig. 1:**
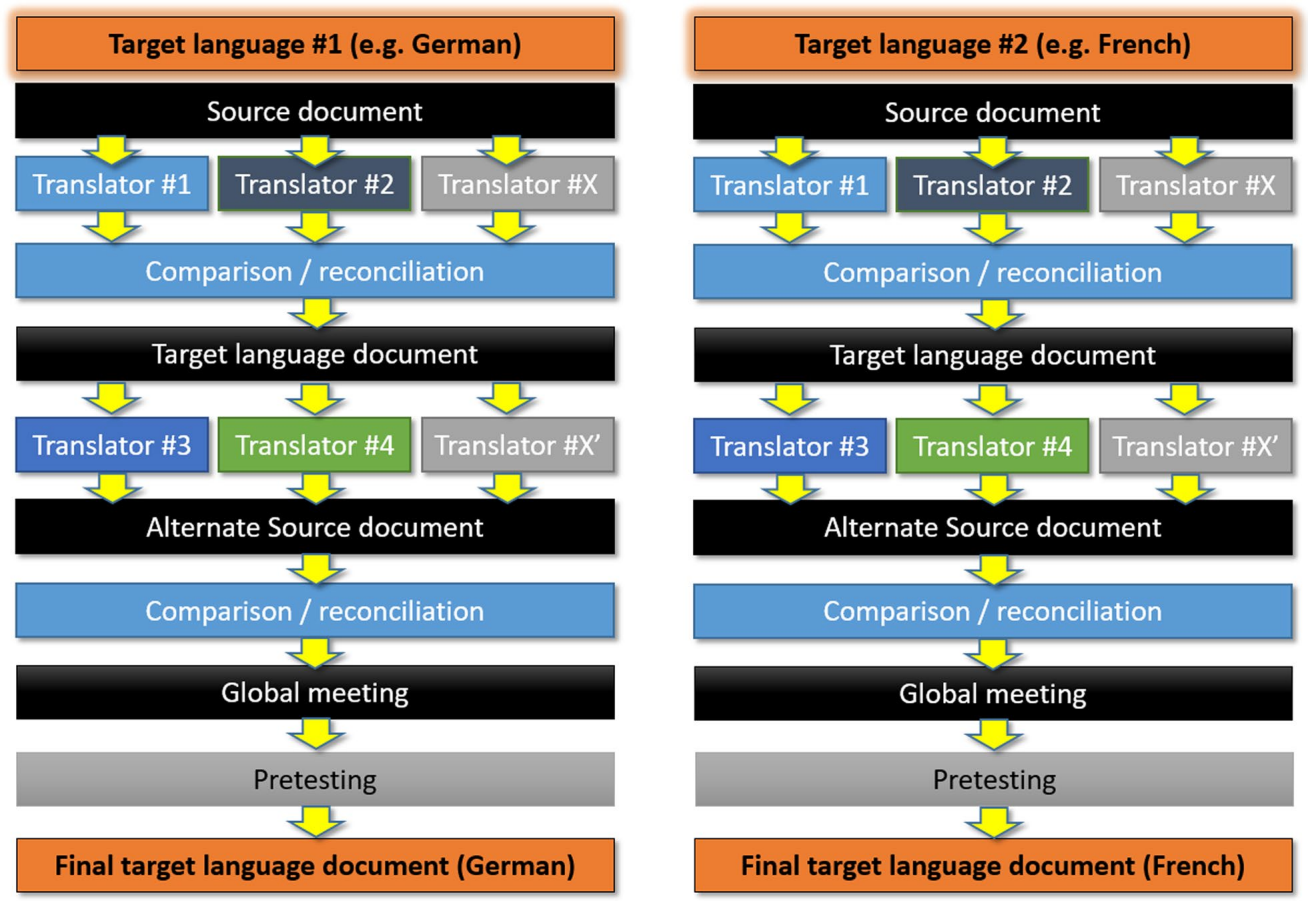
Overview of the classical cross-cultural adaption process for 2 languages

Typically, cross-cultural adaptation involves a systematic (Fig. 1) and collaborative effort which requires input from qualified translators, clinicians, and patients. Although many recommendations have been published, four main stages generally stand out, the details and importance of which vary from one author to another. These main steps include initial forward-translations of the source document by bilingual professionals to render the instrument accurately in the *target language*, while taking into account cultural differences [5]. Then, a group of experts assesses the forward-translated versions, examining their conceptual equivalence, cultural relevance and clarity. Any discrepancies are resolved by reconciliation [6], often through discussion and consensus meeting among the experts. The translated instrument is then back-translated by another group of bilingual professionals to verify accuracy and fidelity to the original language [7]. This stage is controversial in terms of its final content validity and how it is carried out [8, 9]. Subsequently, a Committee review appears constantly in all the proposed methods, as a way to verify the cultural equivalence of the source and final documents. Finally, the pre-(pilot) testing, involves applying the final document to a group of heterogeneous individuals from the target population; the aim of this stage is to identify problems of acceptability or content validity that may not have been identified during the translation stage. This step can be particularly challenging in rare diseases, due to the small number of patients involved.

### General methodology for ReCONNET-CROSSADAPT

The general methodology for ReCONNET-CROSSADAPT, was proposed and drafted by one of the authors (L.A.) through discussions with the RP-QoL steering committee, a team of methodologists (H.D. and T.T) with significant expertise in cross-cultural adaptation of instruments.

Our CROSSADAPT method covers the most important stages of the process: forward translation of the original version by several translators, an expert committee comprising patients and all participants in the previous stages, and pre-testing of the final version with patients. The back-translation stage brings logistical difficulties and is not recommended by all authors [9]. Moreover, studies have shown that the expert committee meeting has a far greater impact on the quality of the final instrument than the back-translation stage [9].

**Fig. 2:**
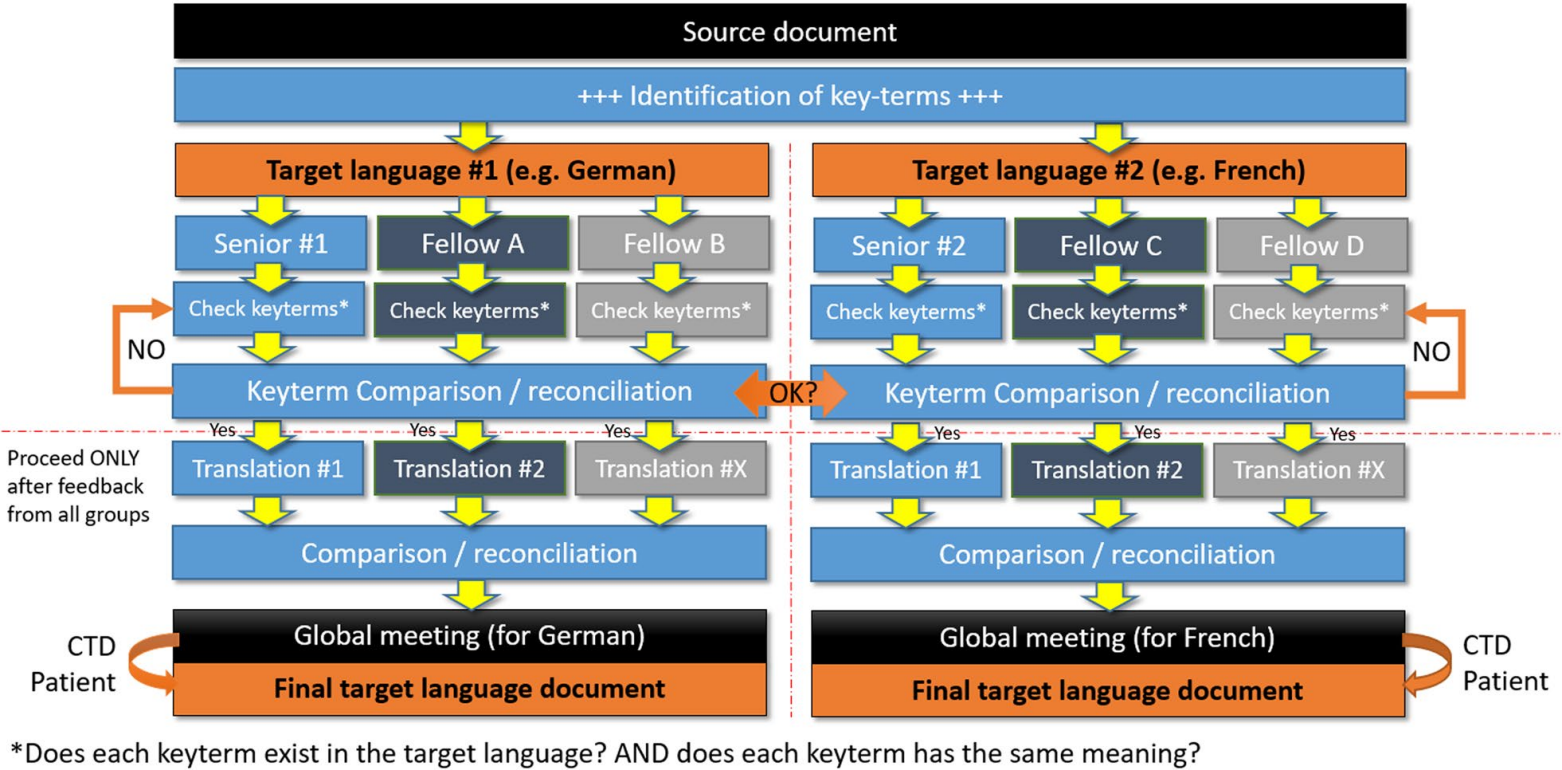
Overview of the ReCONNET-CROSSADAPT methodology for the cross-cultural adaptation of instruments for research and care in the context of rare connective tissue diseases

The ReCONNET-CROSSADAPT process involves several steps (Fig. 2). In short, the first step aims at identifying and verifying *key-terms* (cf. Table 1), the model underlying the questionnaire, its design and content, and the target audience in all language groups involved in the adaptation process. This innovative step recognizes the central role of *key-terms* in ensuring linguistic and conceptual equivalence between different language versions of the document. Step 2 consists of translation by non-specialists, who produce a first version of the translation following a focus group led by the coordinator. During step 3, a reconciliation meeting, acting as a focus group, validates the final version of the translation. The pre-testing stage is then carried out at a later date in each language, prior to the psychometric validation stage.

**Table 1:** Glossary for the ReCONNET-CROSSADAPT

ReCONNET-CROSSADAPT terminology	Detailed meaning
Source document	Document containing the instrument to be cross-culturally adapted, redacted in the *original language*
Original language	Language of the *source document* (e.g. English)
Target language	Language into which the instrument will be cross-culturally adapted (e.g. German)
Key-term	Term or concept from the *source document*, which has been identified by the steering committee as a crucial concept for the cross-cultural adaptation process
Key-term document	Document prepared by the steering committee, containing all *key-terms* and columns to report any adaptation problem from the *source language* to the *target language*
Target disease	CTD(s) about which the documents are related to (e.g. Relapsing polychondritis)
Forward-translation	Translation from the *original language* to the *target language*
Reconciliation Meeting	Meeting convened by the senior member of each language group, to discuss and resolve discrepancies among the interpretation of *key-terms* and translations provided by the collaborators
Tracking document	Document into which all versions, translations, discrepancies and resolution strategies will be recorded
C1 language proficiency level	Corresponds to a user level which can understand long and demanding texts and grasp implicit meanings, speak spontaneously and fluently without having to search for words, use language effectively and flexibly in social, professional or academic life, express on complex subjects in a clear and well-structured way and demonstrate control of the linguistic tools of organization, articulation and cohesion of the discourse
C2 language proficiency level	Corresponds to a user level which can effortlessly understand virtually everything that is read or heard, be able to convey facts and arguments from various written and oral sources in a coherent manner, express spontaneously, very commonly, accurately, and make distinct nuances of meaning in relation to complex subjects

A glossary was developed (Table 1) to provide clear definitions for the main terminology relevant to the cross-cultural adaptation process, ensuring that all involved participants have a common understanding of the terminology used, and facilitating clear communication, comprehensibility, and reproducibility throughout the multilingual adaptation process.

**The preliminary step of ReCONNET-CROSSADAPT is to form a language group for each target language**, each headed by a senior member and made up of two collaborators. These collaborators are usually colleagues, fellows or residents, who meet the following conditions: firstly, they must be native speakers of the *target language*; secondly, they must have a satisfactory level of knowledge in the *original language*, ideally a minimum of C1, preferably C2 (cf. glossary), according to language proficiency standards; thirdly, they must demonstrate a general understanding of medical knowledge related to the *target disease* taken into account in the intercultural adaptation process. By bringing together language groups whose members meet these criteria, the adaptation process ensures adequate linguistic expertise, cultural understanding and medical competence, providing a solid background for successful instrument adaptation across multiple languages and cultures. Of note, additional participants outside the medical field, such as family members or other participants, can be included in the language group, if needed, as long as the core set of a senior member and two collaborators are included.

Each language group must then identify a patient with the *target disease* who meets the following conditions: firstly, this patient must be a native speaker of the *target language*; secondly, must have a satisfactory level of knowledge in the *original language*, ideally at least C1, preferably C2. Following adequate consent, this patient will be involved during the last step of the process (step 3, cf. below). By including a patient who meets these criteria, the adaptation team obtains valuable first-hand information about the patient's perspective, experiences and understanding of the *target disease*. This patient input further reinforces the cultural relevance and patient-centeredness of the adapted document, ensuring that it effectively addresses the needs and concerns of the target population.

**The first step of ReCONNET-CROSSADAPT is the review of the source document for *key-terms***, by the project steering committee. This involves identifying *key-terms* within the *source document* and subsequently highlighting and extracting them. Once this initial step is completed, the *key-term document* is prepared. The *key-term document* contains the original document, all *key-terms*, and columns to report any adaptation problem from the *source language* to the *target language*.

Each language group is then tasked with addressing two critical questions regarding these key-terms: firstly, whether all the identified *key-terms* exist in the *target language*, ensuring linguistic equivalence; and secondly, whether these *key-terms* are interpreted similarly in the *target language*, ensuring conceptual equivalence. If the language group encounters difficulties with one of the *key-terms*, a *reconciliation meeting* becomes necessary. During this meeting, the group collectively assesses whether the identified cross-cultural issue exists and, if confirmed, collaboratively proposes a mitigation strategy. This strategy may involve reaching consensus on a specific term or expression in the *target language* that those language group members agree to use when translating from the *source* to the *target language*. The aim of this approach is to ensure the uniformity and clarity of the translated document, in order to improve understanding and cultural appropriateness in all linguistic contexts.

At this stage, any discrepancies identified in the *key-terms* of one language group are reported to all other language groups for additional verification. This collaborative approach is crucial to facilitate alignment across all *target language* versions of the *source document*. By sharing discrepancies between language groups, the adaptation process encourages collective problem-solving and promotes consistency in the interpretation and adaptation of *key-terms*. This iterative verification process reinforces the reliability and accuracy of the adapted document, reducing the risk of misinterpretations or inconsistencies across different linguistic and cultural contexts.

Altogether, assessing *key-terms* in their cross-cultural perspective serves as a foundational stage in the adaptation process, ensuring that the adapted document accurately captures the intended meaning, and maintains coherence across various linguistic and cultural settings. By identifying and verifying *key- terms*, the new ReCONNET-CROSSADAPT methodology fosters greater collaboration and alignment between adaptation group members. Once all language groups have checked for potential discrepancies in *key-terms* reported by the other language groups, and any potential issue has been resolved, the adaptation process can move forward to the second step.

**During the second step, each member of each language group independently translates the *original document* into the *target language***. Once the translation is complete, the most experienced member of each language group is responsible for organizing a *reconciliation meeting* with the two collaborators of the same language group. During this meeting, any discrepancies identified between the three translations are thoroughly discussed, and efforts are made to reach a consensus on the most appropriate translation for each term or phrase. It is imperative that all discrepancies are resolved by consensus, and that discussions and decisions taken during the reconciliation process are carefully documented in the *tracking document*, facilitating transparency and accountability throughout the adaptation process.

**In the third step of the adaptation process**, the most senior member of each language group, together with the two collaborators and the patient representative of the same language group, address the following two key questions: Does the reconciled translation accurately reflect the content and meaning of the source document? Are all elements of the final reconciled translation appropriate, relevant, clear and understandable? Each member of the adaptation team provides input and comments on these questions, ensuring a comprehensive assessment from a linguistic, cultural and patient perspective. The answers to these questions are recorded in the *tracking document*. By engaging in this collaborative evaluation, the adaptation team ensures that the adapted document remains faithful to the original content, while meeting the linguistic, cultural and communication needs of the target population. If needed, additional and final changes can be made at that stage to meet the aforementioned criteria and agreed upon by consensus by the senior member of each language group, together with the two collaborators and the patient representative.

**In conclusion,** the initial step of the ReCONNET-CROSSADAPT methodology is identifying and verifying *key-terms* across all language groups, combined with a collaborative feedback mechanism. This methodology is suited to the context of rare CTDs, which are characterized by limited resources and the need for culturally adapted tools. This innovative approach may offer a flexible adaptation framework for guaranteeing linguistic accuracy, cultural relevance and contextual appropriateness., and is currently used within the general framework of the ERN ReCONNET, for the cross-cultural adaptation of several research and care instruments for rare diseases, including a disease-specific health-related quality of life instrument for relapsing polychondritis, the development of a multilingual item bank for testing the essential knowledge of patients with autoimmune diseases such as lupus, as well as for the development of an instrument aiming at identifying patient at risk of vulnerabilities during medical care.

## Supplementary Information

Additional file 1.

## Data Availability

All data supporting the findings of this study are available within the paper.
