# Supplementary material for: European Reference Network (ERN) ReCONNET methodology for the cross-cultural adaptation of instruments for research and care in the context of rare connective tissue diseases (CROSSADAPT)
Source: Orphanet J Rare Dis. 2025 May 15;20:230. doi: 10.1186/s13023-025-03674-8 (PMC12080175; doi:10.1186/s13023-025-03674-8)
Supplement: Supplementary file 1 — Additional file 1. [file 13023_2025_3674_MOESM1_ESM.pdf]

**European Reference Network (ERN) ReCONNET methodology for the cross-cultural adaptation of instruments for research and care in the context of rare connective tissue diseases (CROSSADAPT)**

*The traditional process of intercultural adaptation, while suitable for one or a few target languages, is not optimal for developing instruments for rare connective tissue diseases (CTDs) in multiple languages simultaneously. The ERN ReCONNET presents a novel methodology for cross-cultural adaptation of research and care instruments in the context of rare CTDs (ReCONNET-CROSSADAPT).*

**Summary of the ReCONNET-CROSSADAPT process:**

The ReCONNET-CROSSADAPT methodology involves several steps. The first step aims at identifying and verifying *key-terms*, ensuring linguistic and conceptual equivalence between different language versions of the source document. Step 2 consists of translation by non-specialists, who produce a first version of the translation following a focus group led by the coordinator. During step 3, a reconciliation meeting, acting as a focus group, validates the final version of the translation.

**Visual summary of the ReCONNET-CROSSADAPT process:**

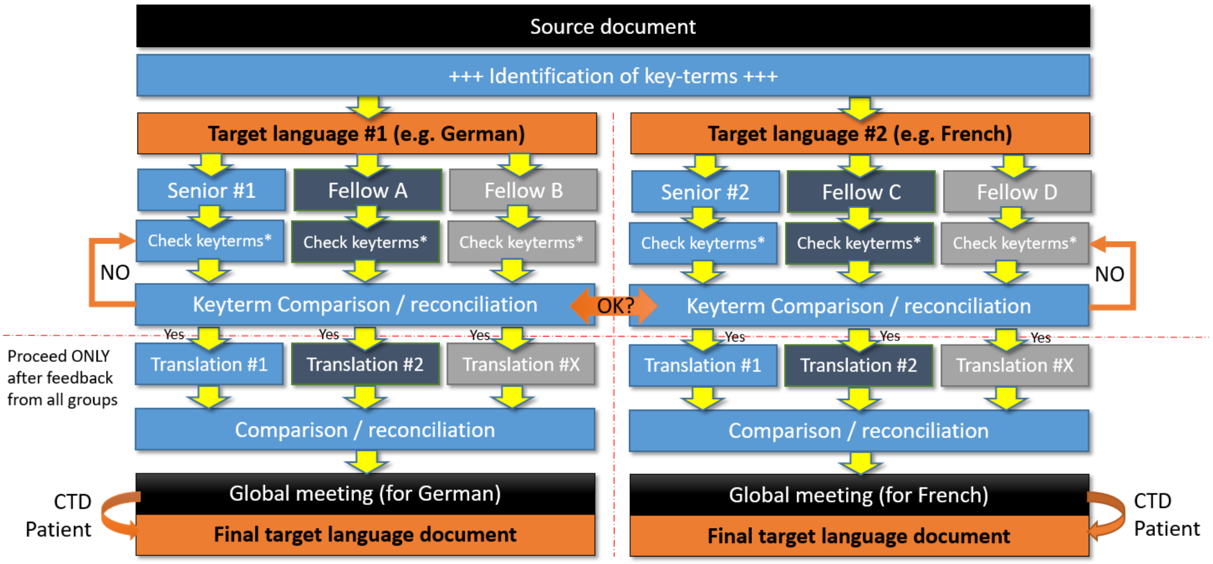


*Does each keyterm exist in the target language? AND does each keyterm has the same meaning?

**In the example shown below,** a group of international investigators from the ERN ReCONNET disease group for Relapsing polychondritis, a rare systemic inflammatory disease involving the cartilages as well as various other organs, conveyed to adapt the script of an online patient survey, from English (original language) to 5 target languages: German, French, Italian, Portuguese and Romanian. This patient survey was part of a broader research project, aiming at the development of a disease-specific health related quality of life instrument, the RP-QoL.

***The preliminary step of ReCONNET-CROSSADAPT is to form a language group for each target language****, each headed by a senior member and made up of two collaborators.*

In the aforementioned example, the steering committee of the RP-QoL Study assembled 5 language groups (one for each target language), involving one language coordinator and 2 collaborators. Each of these 15 individuals had satisfactory level of knowledge in the *original language (English)*, and

demonstrated a general understanding of medical knowledge related to the *target disease (relapsing polychondritis)* taken into account in the intercultural adaptation process. Each language then identified a relapsing polychondritis patient who was a native speaker of the *target language* and had a satisfactory level of knowledge in English.

***The first step of ReCONNET-CROSSADAPT is the review of the source document for key-terms****, by the project steering committee.*

During that step, the steering committee met online, and reviewed by consensus the script of the survey (cf source document, below), and extracted key-terms (highlighted in yellow)

**Source document for the RP-QoL Survey**

| | |
|---|---|
| Question A1 | **Please list the areas of your life (considering all aspects) that are impacted by relapsing polychondritis**<br>- *Feel free to mention as many elements as you wish*<br>- *There is no need to provide details at that stage, as we will ask you more specific questions during the remaining of the interview*<br>- *Please note that you will not be able to come back to this question once you have clicked 'next'.* |
| **Now we would like to understand more precisely the impact of relapsing polychondritis upon several domains of life**<br>- *Please note that this section contains a total of 10 questions (so that you can manage your time!)*<br>- *When answering, please try to explain in your own words what is the impact of relapsing polychondritis upon the domain considered, and how it affects your life*<br>- ***When possible, try to avoid very short answers such as: 'yes', 'no', 'no impact', or 'a lot'*** | |
| Question B1 | How would you describe the impact of relapsing polychondritis on your **daily life and activities**? |
| Question B2 | How would you describe the impact of relapsing polychondritis on your **professional life** (**or studies**, if you are a student)? |
| Question B3 | How would you describe the impact of RP on **relationships (family, intimate life, friends, etc…)** |
| Question B4 | How would you describe the impact of relapsing polychondritis on your **body**? |
| Question B5 | How would you describe the impact of relapsing polychondritis on the way **you see yourself?** |
| Question B6 | How would you describe the impact of relapsing polychondritis **on your mind/cognition** (e.g., thinking, learning, memory and concentration, etc…) |
| Question B7 | How would you describe the impact of relapsing polychondritis on your **energy and sleep**? |
| Question B8 | How would you describe the impact of **medications and non-pharmacological treatments** for relapsing polychondritis upon your life? |
| Question B9 | Despite the impacts described in previous questions, did you identify any **positive impact** of relapsing polychondritis upon your life (e.g., learnings or personal growth)? Can you describe it? |
| Question B10 | **Is there anything else that you would like to comment on that we have not already asked you about?** |

Once this initial step was completed, the *key-term document* was prepared. The *key-term document* contained the original document, all *key-terms*, and columns to report any adaptation problem from the *source language* to the *target language*.

## Example of key-term document for the RP-QoL study

| keyterm number | Question Nb | Questions | EXTRACTED KEYTERM | Rater 1. The keyterm EXISTS in the target languge? (YES or NO) | Rater 1. The keyterm is INTERPRETED SIMILARLY in the target languge? (YES or NO) | Rater 2. The keyterm EXISTS in the target languge? (YES or NO) | Rater 2. The keyterm is INTERPRETED SIMILARLY in the target languge? (YES or NO) | Rater 3. The keyterm EXISTS in the target languge? (YES or NO) | Rater 3. The keyterm is INTERPRETED SIMILARLY in the target languge? (YES or NO) |
|---|---|---|---|---|---|---|---|---|---|
| 1 | Question A1 | Please list the areas of your life (considering all aspects) that are impacted by relapsing | areas of your life | | | | | | |
| 2 | Question A1 | Please list the areas of your life (considering all aspects) that are impacted by relapsing | impact/impacted | | | | | | |
| 3 | Question B1 | How would you describe the impact of relapsing polychondritis on your daily life and activities? | daily life | | | | | | |
| 4 | Question B1 | How would you describe the impact of relapsing polychondritis on your daily life and activities? | activities | | | | | | |
| 5 | Question B2 | How would you describe the impact of relapsing polychondritis on your professional life (or studies, if | professional life | | | | | | |
| 6 | Question B2 | How would you describe the impact of relapsing polychondritis on your professional life (or studies, if | studies / student | | | | | | |
| 7 | Question B3 | How would you describe the impact of RP on relationships (family, intimate life, friends, etc...) | relationships | | | | | | |
| 8 | Question B3 | How would you describe the impact of RP on relationships (family, intimate life, friends, etc...) | family | | | | | | |
| 9 | Question B3 | How would you describe the impact of RP on relationships (family, intimate life, friends, etc...) | intimate life | | | | | | |
| 10 | Question B3 | How would you describe the impact of RP on relationships (family, intimate life, friends, etc...) | friends | | | | | | |
| 11 | Question B4 | How would you describe the impact of relapsing polychondritis on your body? | body | | | | | | |
| 12 | Question B5 | How would you describe the impact of relapsing polychondritis on the way you see yourself? | you see yourself | | | | | | |
| 13 | Question B6 | How would you describe the impact of relapsing polychondritis on your mind/cognition | mind | | | | | | |
| 14 | Question B6 | How would you describe the impact of relapsing polychondritis on your mind/cognition | cognition | | | | | | |
| 15 | Question B7 | How would you describe the impact of relapsing polychondritis on your energy and sleep? | Energy | | | | | | |
| 16 | Question B7 | How would you describe the impact of relapsing polychondritis on your energy and sleep? | Sleep | | | | | | |
| 17 | Question B8 | How would you describe the impact of medications and non-pharmacological treatments for relapsing | medications | | | | | | |
| 18 | Question B8 | How would you describe the impact of medications and non-pharmacological treatments for relapsing | on-pharmacological treatment | | | | | | |
| 19 | Question B9 | Despite the impacts described in previous questions, did you identify any positive impact of | positive impact | | | | | | |
| 20 | Question B10 | Is there anything else that you would like to comment on that we have not already asked you about? | *No keyterm* | | | | | | |

Each of the 5 language groups was then tasked with addressing two critical questions regarding these key-terms: firstly, whether all the identified *key-terms* exist in the *target language*, ensuring linguistic equivalence; and secondly, whether these *key-terms* are interpreted similarly in the *target language*, ensuring conceptual equivalence.

*If the language group encountered difficulties with one of the key-terms, a reconciliation meeting became necessary. During this meeting, the group collectively assesses whether the identified cross-cultural issue exists and, if confirmed, collaboratively proposes a mitigation strategy. This strategy may involve reaching consensus on a specific term or expression in the target language that those language group members agree to use when translating from the source to the target language. The aim of this approach is to ensure the uniformity and clarity of the translated document, in order to improve understanding and cultural appropriateness in all linguistic contexts.*

In the RP-QoL survey example, participants from the German group highlighted 3 key-terms, for which a cross-cultural issue might exist and, collaboratively proposed a mitigation strategy (ie. one agreed-upon translation for each of these 3 key-words)

| EXTRACTED KEYTERM | Rater 1. The keyterm EXISTS in the target languge? (YES or NO) | Rater 1. The keyterm is INTERPRETED SIMILARLY in the target languge? (YES or NO) | Rater 2. The keyterm EXISTS in the target languge? (YES or NO) | Rater 2. The keyterm is INTERPRETED SIMILARLY in the target languge? (YES or NO) | Rater 3. The keyterm EXISTS in the target languge? (YES or NO) | Rater 3. The keyterm is INTERPRETED SIMILARLY in the target languge? (YES or NO) | pb confirmed after discussion | proposed mitigation strategy |
|---|---|---|---|---|---|---|---|---|
| areas of your life | YES | YES | Yes | Yes | yes | yes | | |
| impact/impacted | YES | YES | Yes | Yes | yes | yes | | |
| daily life | YES | YES | Yes | Yes | yes | yes | | |
| activities | YES | YES | Yes | Yes | yes | yes | | |
| professional life | YES | YES | Yes | Yes | yes | yes | | |
| studies / student | YES | YES | Yes | Yes | yes | yes | | |
| relationships | YES | YES | Yes | Yes | yes | yes | | |
| family | YES | YES | Yes | Yes | yes | yes | | |
| intimate life | YES | YES | No | Yes | yes | yes | yes | Intimleben |
| friends | YES | YES | Yes | Yes | yes | yes | | |
| body | YES | YES | Yes | Yes | yes | yes | | |
| you see yourself | YES | YES | Yes | Yes | yes | yes | | |
| mind | YES | no | Yes | No | yes | no | yes | Gedanken |
| cognition | YES | no | Yes | No | yes | no | yes | Wahrnehmung |
| Energy | YES | YES | Yes | Yes | yes | yes | | |
| Sleep | YES | YES | Yes | Yes | yes | yes | | |
| medications | YES | YES | Yes | Yes | yes | yes | | |
| Non-pharmacological treatment | YES | YES | Yes | Yes | yes | yes | | |
| positive impact | YES | YES | Yes | Yes | yes | yes | | |
| *No keyterm* | | | | | | | | |

*At this stage, any discrepancies identified in the key-terms of one language group were reported to all other language groups for additional verification. This collaborative approach was crucial to facilitate alignment across all target language versions of the source document. By sharing discrepancies between language groups, the adaptation process encouraged collective problem-solving and promotes consistency in the interpretation and adaptation of key-terms.*

In the RP-QoL example, none of the keywords identified as problematic by any of the language group was cross-reported at this stage as problematic by the other language group.

*Once all language groups had checked for potential discrepancies in key-terms reported by the other language groups, and any potential issue had been resolved, the adaptation process was able to move forward to the second step.*

**During the second step, each member of each language group independently translates the *original document* into the *target language*.**

An extract of the 3 translations to German is shown below. Although relatively similar, the 3 translations contained discrepancies (discrepancies for the 1st question are shown in red).

**Example of the 3 German translations, for the RP-QoL study**

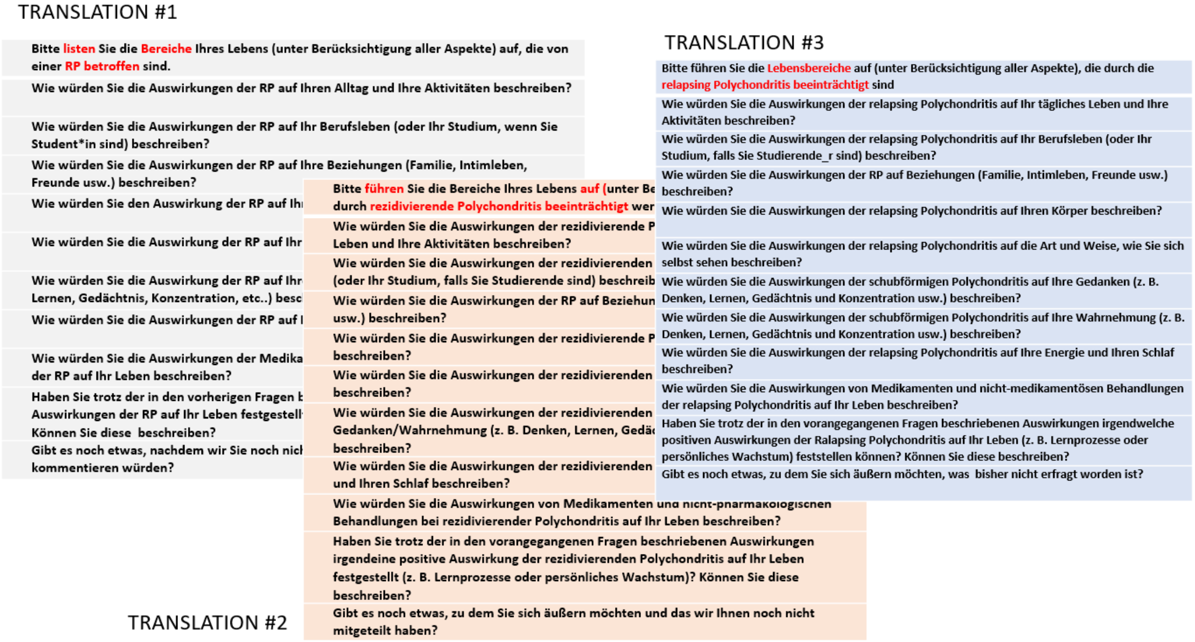


Once the translation is complete, the most experienced member of each language group was responsible for organizing a *reconciliation meeting* with the two collaborators of the same language group. During this meeting, any discrepancies identified between the three translations were thoroughly discussed, and efforts are made to reach a consensus on the most appropriate translation for each term or phrase.

The final, reconciliated, German adaptation for the RP-QoL survey script is shown below

## FINAL (RECONCILIATED GERMAN ADAPTATION)

| Questions | Fragen |
|---|---|
| Please list the areas of your life (considering all aspects) that are impacted by relapsing polychondritis | Bitte führen Sie die Lebensbereiche auf (unter Berücksichtigung aller Aspekte), die durch die Relapsing Polychondritis beeinträchtigt sind |
| How would you describe the impact of relapsing polychondritis on your daily life and activities? | Wie würden Sie die Auswirkungen der Relapsing Polychondritis auf Ihren Alltag und Ihre Aktivitäten beschreiben? |
| How would you describe the impact of relapsing polychondritis on your professional life (or studies, if you are a student)? | Wie würden Sie die Auswirkungen der Relapsing Polychondritis auf Ihr Berufsleben (oder Ihr Studium, falls Sie Studierende oder Studierender sind) beschreiben? |
| How would you describe the impact of RP on relationships (family, intimate life, friends, etc...) | Wie würden Sie die Auswirkungen der Relapsing Polychondritis auf Ihre Beziehungen (Familie, Intimleben, Freunde usw.) beschreiben? |
| How would you describe the impact of relapsing polychondritis on your body? | Wie würden Sie die Auswirkungen der Relapsing Polychondritis auf Ihren Körper beschreiben? |
| How would you describe the impact of relapsing polychondritis on the way you see yourself? | Wie würden Sie die Auswirkungen der Relapsing Polychondritis auf Ihr Selbstbild beschreiben? |
| How would you describe the impact of relapsing polychondritis on your mind/cognition (e.g., thinking, learning, memory and concentration, etc...) | Wie würden Sie die Auswirkungen der Relapsing Polychondritis auf Ihre Gedanken und Wahrnehmung (z. B. Denken, Lernen, Gedächtnis und Konzentration usw.) beschreiben? |
| How would you describe the impact of relapsing polychondritis on your energy and sleep? | Wie würden Sie die Auswirkungen der Relapsing Polychondritis auf Ihre Energie und Ihren Schlaf beschreiben? |
| How would you describe the impact of medications and non-pharmacological treatments for relapsing polychondritis upon your life?" | Wie würden Sie die Auswirkungen von Medikamenten und nicht-medikamentösen Behandlungen der Relapsing Polychondritis auf Ihr Leben beschreiben? |
| Despite the impacts described in previous questions, did you identify any positive impact of relapsing polychondritis upon your life (e.g., learnings or personal growth)? Can you describe it? | Haben Sie trotz der in den vorangegangenen Fragen beschriebenen Auswirkungen positive Auswirkungen der Relapsing Polychondritis auf Ihr Leben (z. B. Lernprozesse oder persönliche Entwicklung) feststellen können? Können Sie diese beschreiben? |
| Is there anything else that you would like to comment on that we have not already asked you about? | Gibt es noch etwas, nachdem wir Sie noch nicht gefragt haben und das Sie gerne kommentieren würden? |

***In the third step of the adaptation process**, the most senior member of each language group, together with the two collaborators and the patient representative of the same language group, addressed the following two key questions:*

- *Does the reconciled translation accurately reflect the content and meaning of the source document?*
- *Are all elements of the final reconciled translation appropriate, relevant, clear and understandable?*

Each member of the adaptation team provided input and comments on these questions, ensuring a comprehensive assessment from a linguistic, cultural and patient perspective.

**Example of final feedback from the German language group, regarding the adaptation of the RP-QoL survey from English to German:**

C. Together with the 2 collaborators and the patient (this can be asynchronous), answer the following questions (record answers into the *tracking document*) ❑

a. Does the *reconciliated forward-translation* **reflects the *source document* content** and **sense**?
X *Yes or* ❑ *No*

b. Are all items from the *reconciliated forward-translation* **suitable, relevant, clear and comprehensible**
X *Yes or* ❑ *No*

**These steps were conducted simultaneously and in parallel for the 5 target languages.**
